# High‐resolution, relational, resonance‐based, electroencephalic mirroring (HIRREM) improves symptoms and autonomic function for insomnia: A randomized, placebo‐controlled clinical trial

**DOI:** 10.1002/brb3.1826

**Published:** 2020-09-17

**Authors:** Catherine L. Tegeler, Hossam A. Shaltout, Sung W. Lee, Sean L. Simpson, Lee Gerdes, Charles H. Tegeler

**Affiliations:** ^1^ Department of Neurology Wake Forest School of Medicine (WFSM) Winston‐Salem NC USA; ^2^ Hypertension and Vascular Research Center WFSM Winston‐Salem NC USA; ^3^ University of Arizona School of Medicine Phoenix AZ USA; ^4^ Department of Biostatistics and Data Sciences WFSM Winston‐Salem NC USA; ^5^ Brain State Technologies, LLC Scottsdale AZ USA

**Keywords:** acoustic neuromodulation, allostasis, autonomic, closed‐loop neurotechnology, HIRREM, insomnia

## Abstract

**Introduction:**

Effective insomnia interventions that also address autonomic dysregulation are lacking. We evaluate high‐resolution, relational, resonance‐based, electroencephalic mirroring (HIRREM^®^), in a randomized, controlled clinical trial. HIRREM is a noninvasive, closed‐loop, allostatic, acoustic stimulation neurotechnology, to support self‐optimization of brain rhythms.

**Methods:**

One hundred and seven adults (mean age 45.7, *SD* ± 5.6, 73 women), with Insomnia Severity Index (ISI) scores of ≥15, received ten, 90‐min sessions of HIRREM, with tones linked to brainwaves (LB, 56), or random tones not linked to brainwaves (NL, 51), as an active, sham placebo. Outcomes were obtained at enrollment (V1), 1–7 days (V2), 8–10 weeks (V3), and 16–18 weeks (V4) after intervention. Primary outcome was differential change in ISI from V1 to V3. Secondary measures assessed depression (BDI), anxiety (BAI), quality of life (EQ‐5D), and a sleep diary. Ten minute recordings of HR and BP allowed analysis of heart rate variability (HRV) and baroreflex sensitivity (BRS).

**Results:**

Of 107 randomized, 101 completed the intervention. Intention‐to‐treat analysis (107) of change from V1 to V3 revealed a mean reduction of ISI in NL of −4.93 (*SE* ± 0.76) points, with additional, significant reduction of −2.05 points (0.74) in LB (total reduction of −6.98, *p* = .045). Additional reduction of −2.30 points (0.76) was still present in the LB at V4 (*p* = .058). Total ISI reduction from V1 to V4 was −5.90 points for NL and −7.93 points in LB. There were group differences (*p* < .05) for multiple HRV and BRS measures (rMSSD, SDNN, HF alpha, and Seq ALL), as well as total sleep time, sleep onset latency, and sleep efficiency. There were no serious adverse events.

**Conclusions:**

Results of this controlled clinical trial showed clinically relevant reduction of insomnia symptoms with HIRREM, over, and above an active, sham control, with associated, durable improvement in autonomic cardiovascular regulation.

## Graphical abstract

This controlled clinical trial observed significant added benefit to reduce symptoms of insomnia with use of a closed‐loop, acoustic stimulation neurotechnology that echoes tones linked to brainwaves (HIRREM), compared to a sham using randomly generated tones. There was also significant improvement in multiple measures of autonomic cardiovascular regulation, with durability to 4 months postintervention, and group differences for sleep efficiency, total sleep time, and sleep onset latency. Results suggest that this noninvasive, nondrug intervention could provide a viable alternative for moderate to severe insomnia.

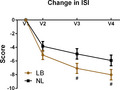

## 1 INTRODUCTION

Insomnia is a public health problem and is recognized for its significance related to behavioral disorders, risk factor for physical diseases, and hazard to occupational performance (Riemann et al., 2017). The DSM‐5 removed the distinction between primary and secondary forms to support emphasis that insomnia should be a target for treatment, regardless of etiology (Association & A. P., 2013). Numerous epidemiological studies show that self‐reported insomnia symptoms or circadian rhythm disruptions are a risk factor for dysregulation or adverse outcomes in the cardiovascular, metabolic, neurological, and other organ systems, as well as mortality (Wulff, Gatti, Wettstein, & Foster, 2010; Young & Bray, 2007). Insomnia also contributes to reduction in worker productivity, and increased absenteeism and accident risk (Daley et al., 2009; Espie et al., 2018; Kucharczyk, Morgan, & Hall, 2012).

Though cognitive behavioral therapy is established as an efficacious first line intervention (Riemann et al., 2017), and psychopharmacological treatments are frequently used (Riemann et al., 2017; Trauer, Qian, Doyle, Rajaratnam, & Cunnington, 2015), there is ample room for innovation in insomnia therapy. Many individuals are not good candidates for current therapies due to side effects, risk for dependence, time constraints, personal preference, or lack of efficacy. Furthermore, in light of demonstrated relationships between insomnia and physical health disruptions, an open question is whether or how the treatment of insomnia may generate benefits for other organ systems. For example, although a clinical trial of an Internet‐based sleep support intervention for individuals with mild sleep impairment and mild to moderate hypertension did not show an effect for blood pressure reduction, the authors considered whether their null finding may have been due to selection factors or length of follow‐up (McGrath et al., 2017).

The inclusion of outcome measures which reflect functioning of physiological regulatory pathways may strengthen the inferences that can be made from clinical studies of insomnia treatments. Heart rate variability (HRV) is a metric which indicates the relative contribution of sympathetic versus parasympathetic influences in autonomic regulation (Malik & Task Force for the European Society of Cardiology and The North American Society of Pacing Electrophysiology, 1996), and prospective studies show that lower levels of HRV are associated with increased cardiovascular and all‐cause mortality (Dekker et al., 1997; Kleiger, Miller, Bigger, & Moss, 1987). Reduction of measures of HRV has been observed with insomnia (Farina et al., 2014; Jurysta et al., 2009) and is consistent with the hyperarousal theory, which has now been supported by polysomnographic and neuroimaging findings (Hein et al., 2017; Levenson, Kay, & Buysse, 2015; O'Byrne, Berman Rosa, Gouin, & Dang‐Vu, 2014; Riemann et al., 2010).

Heart rate variability may thus have applicability as a surrogate indicator for the adverse effects of insomnia on the cardiovascular system (Jarrin et al., 2018; Nano, Fonseca, Vullings, & Aarts, 2017). Moreover, there are two studies which suggest that improvement in HRV may be a marker of response to true therapy for insomnia (Campana, Clifford, Trinder, Pittman, & Malhotra, 2011; Chung, An, Park, & Kim, 2011).

In recent years, closed‐loop technologies have been explored as a precision‐guided way to impact neural circuits associated with mental health or behavioral disorders (Lo & Widge, 2017; Mishra & Gazzaley, 2014). Through repeated cycles of real time monitoring and calibrated intervention, closed‐loop neurotechnologies have the potential to evaluate an individual's unique and changing patterns of brain activity and to make dynamic therapeutic adjustments within time frames of milliseconds. High‐resolution, relational, resonance‐based electroencephalic mirroring (HIRREM^®^, registered trademark of Brain State Technologies) is a closed‐loop, acoustic stimulation, neurotechnology based on the principle of allostasis (Gerdes, Gerdes, Lee, & Tegeler, 2013). HIRREM was found to be associated with reduction of both insomnia symptomatology neurophysiological arousal in a pilot clinical trial which included a waiting‐list control group (Tegeler et al., 2012). Reduced insomnia symptoms and improved autonomic cardiovascular regulation were observed in a large open label series (Shaltout et al., 2018). Reduced self‐reported symptoms of insomnia, PTSD, depression, and anxiety, improved autonomic cardiovascular regulation, and significant changes in network connectivity on whole brain rest MRI, was reported in a cohort with military‐related symptoms of post‐traumatic stress (Lee et al., 2019; Tegeler, Gerdes, et al., 2017).

The objective of the present study was to evaluate the efficacy of HIRREM for individuals with self‐reported symptoms of moderate to severe insomnia, using a larger sample and a placebo‐controlled study design. We hypothesized that usage of closed‐loop, acoustic stimulation linked to brainwaves (HIRREM) would result in reduced symptoms of insomnia and improvements in autonomic cardiovascular regulation, compared to exposure to an active, sham placebo condition consisting of randomly generated tones not linked to brainwaves, given similar levels of social support and sensory stimulation. We now report main clinical and autonomic outcomes.

## 2 METHODS

### 2.1 Study participants

This single site, controlled clinical trial was carried out in the Department of Neurology at Wake Forest Baptist Health, an academic medical center in Winston‐Salem, North Carolina. A total of 694 individuals were assessed for eligibility, and 107 men and women age 18 or older were enrolled (mean age 53.3 ± 14.6, 69 women). Other key demographics are noted in Table 1. Participants had a clinical diagnosis of insomnia not attributable to another know cause, for example, obstructive sleep apnea, restless legs syndrome, or benign prostatic hypertrophy, and a score of ≥15 on the Insomnia Severity Index, and were recruited by community physician referral and advertisement. Potential participants were excluded if they were unable, unwilling, or incompetent to provide informed consent, or physically unable to attend study visits. Other exclusions included a known history of obstructive sleep apnea, diagnosed periodic limb movements disorder, seizure disorder, urinary problems such as benign prostate hypertrophy as the likely cause of sleep disturbance, severe hearing impairment, known or suspected diagnosis of post‐traumatic stress disorder (PTSD), known, relevant traumatic brain injury (TBI), or ongoing need for treatment with opiate, benzodiazepine, or antipsychotic medications, antidepressant medications such as selective serotonin reuptake inhibitors (SSRI), serotonin–norepinephrine reuptake inhibitor (SNRI), or tricyclics, and sleep medications such as zolpidem or eszopiclone. Those with anticipated, ongoing use of recreational drugs or alcohol, or lack of Internet or smart phone access were also excluded. Participants were requested to abstain from using alcohol or recreational drugs during the intervention, and for at least 3 weeks following sessions. Participants were also advised to suspend chiropractic, cranial‐sacral therapy, and bio‐energy work during the intervention, and for at least 3 weeks following, and were asked to refrain from caffeine use after 1:00 p.m. All participants were instructed to continue their current care, which was defined as whatever other medications or therapies, outside of those listed above as exclusions, that subjects were using prior to enrollment.

**Table 1:** Participant demographics

Variable	LB Group (*n* = 56)	NL Group (*n* = 51)
Socio‐demographics		
Age, mean (*SD*), y	52.4 (15.1)	54.7 (14.8)
Female, No. (%)	41 (73.2)	32 (62.7)
White non‐Hispanic race/ethnicity, No. (%)	46 (82.1)	43 (84.3)
Self‐reported comorbidities, No. (%)		
Chronic pain	6 (10.7)	6 (11.8)
Depression	10 (17.9)	12 (23.5)
Diabetes	3 (5.4)	3 (5.9)
Headaches	13 (23.2)	14 (27.4)
Hot flashes	12 (21.4)	11 (21.6)
Hyperlipidemia	6 (10.7)	9 (17.6)
Hypertension	16 (28.6)	10 (19.6)
Migraines	9 (16.1)	7 (13.7)
Stress/anxiety	7 (12.5)	6 (11.8)
Self‐reported sleep characteristics, No. (%)		
Trouble falling asleep	48 (85.7)	47 (92.2)
Trouble staying asleep	56 (100)	51 (100)
Not waking rested	42 (75)	41 (80.4)
Duration with sleep trouble, mean (*SD*), y	11.1 (12.1)	12.2 (11.3)

### 2.2 Study design

A randomized, blinded, placebo‐controlled study design was used. Study participants, as well as all study personnel, except for the Technologists administering the intervention, remained blinded to group assignment. The protocol was approved by the Institutional Review Board at Wake Forest University Health Sciences, which did not require data safety and monitoring board oversight. The 107 participants were randomly allocated based on a blocked randomization, with a block size of 4, and a 1:1 ratio, and form the cohorts used for the intention‐to‐treat analyses. Standard intention‐to‐treat analysis is an approach in which all randomized participants are included in the statistical analyses even if they do not have follow‐up data (McCoy, 2017; Montedori et al., 2011)}. The randomization scheme and assignments used sequentially numbered, sealed envelopes containing group assignment, were created independently by a team member who had no contact with the participants, and were securely maintained by the Chief Technologist. Group assignments were made independent of the team member enrolling the participant. The study was approved to enroll up to 130 participants in order to achieve a goal of at least 100 to complete the intervention.

Fifty‐six participants were assigned to receive the HIRREM intervention, consisting of tones linked to brainwaves (LB), while 51 were assigned to receive random tones not linked to brainwaves (NL), in addition to continued current care. Written informed consent and all baseline measures, along with a brainwave assessment, were obtained during an enrollment visit (V1), and the participant started a daily sleep diary, which was to be maintained until the primary outcome was completed. The period of intervention, either HIRREM or placebo, began 7–14 days following V1. Participants received 10 intervention sessions over a 3‐week period. Participants could receive two intervention sessions during a half day period, with a goal for all to receive a total of 4 sessions during the first 2 days of the intervention period. Participants were encouraged to get the 5th and 6th session during the first week and to complete the remainder of the 10 intervention sessions within 2 weeks. Following completion of the initial 4 sessions, the remainder could be arranged as singles (one per day), if needed, due to schedule issues. Sessions were typically administered based on convenience, and schedule needs for the participant. The time of day was noted for both intervention sessions and data collections.

One to seven days after the final intervention session, there was a postintervention data collection visit (V2). All measures were repeated, but no brainwave assessment was obtained. Eight to ten weeks after completion of the intervention, there was another postintervention data collection visit (V3), which served as the primary outcome for the study so all measures were repeated, and a brainwave assessment obtained. The daily sleep diary was discontinued following V3. A final data collection visit (V4) occurred 16–18 weeks after completion of the intervention, with repeat of the outcome measures, but no brainwave assessment. The blind was broken at the V4 visit, and group assignment shared with the participant. Although official involvement in the study was then completed, those who were in the NL group were offered a chance to receive a course of HIRREM. An expectation measure regarding group assignment LB, or to NL, was obtained at V1, at completion of the 4th intervention session, and at the V3 visit.

### 2.3 Closed‐loop neurotechnology and placebo interventions

#### 2.3.1 Brainwave assessment

As part of the enrollment visit, prior to the initial intervention session, participants received a brainwave assessment. Participants received a repeat brainwave assessment as part of the V3 data collection. Brainwave assessments were performed with the participant in a sitting position. Sensors were sequentially placed over at least six paired locations on the scalp to record 1 min epochs of data while the brain is at rest, or on task, with eyes open and with eyes closed. Measurements were taken at homologous regions of the bilateral hemispheres according to the 10–20 International System (Jasper, 1958) at F3/F4, C3/C4, P3/P4, T3/T4, FZ/OZ, O1/O2, FP1/FP2, and CB1/CB2 with both eyes closed (EC; 1 min), eyes partially open (1 min), and eyes open (EO; 1 min) conditions. For EO assessments, subjects were given standardized tasks involving numerical digit recall (F3/F4), reading silently (C3/C4), math calculations (P3/P4), listening comprehension (T3/T4), and to relax with eyes open (O1/O2). A sixth midline measurement was taken at FZ/OZ, with an EO task to count number of appearances of a specific word as they read a standardized printed passage. The reference sensors were connected at A1/A2 and linked for assessments. The assessment required about 30–45 min to complete.

Assessment data were reviewed by trained Technologists to pick protocols for the first intervention session. HIRREM software algorithms are intended to support de‐establishment of relatively inflexible and possibly maladaptive patterns of activity. Particular attention is given to activity patterns suggesting dominant hemispheric asymmetries and/or suboptimal ratios of electrical amplitudes across the spectrum of frequencies (Gerdes et al., 2013). Best practices were applied to choose protocols. Pilot data also suggest utility for correlation of brain pattern with autonomic cardiovascular regulation outcomes (heart rate variability, HRV) and that changes in asymmetry of frequencies and amplitudes might be observed from pre‐ to post‐HIRREM (Shaltout et al., 2018; Tegeler, Cook, et al., 2017).

#### 2.3.2 HIRREM/Placebo intervention sessions

All participants received 10 intervention sessions of 1.5–2 hr in length, consisting of roughly 4–8 individual protocols, each typically lasting from 6–40 min. During sessions, with the subject comfortably at rest, sitting, or reclining, paired sensors were placed over specific target areas on the scalp corresponding with brain regions/lobes to be observed. For those in the LB group, software algorithms identified specific frequencies and translated them to audible tones in real time. These were echoed back to the participant via ear buds with as little as 4–8 ms delay. Participants were thus able to “listen to their brain” and to figuratively speaking observe their brain pattern in an acoustic mirror, via tones linked in real time to the energetic pattern in the brain.

This closed‐loop, recipient‐unique, acoustic‐stimulation brain feedback, or acoustic neuromodulation, supports the brain to auto‐calibrate, to self‐adjust, to relax, and to shift toward a more balanced state, often with reduced hyperarousal. This, with no need for active, conscious, cognitive involvement by the participant, operant conditioning, or training the brain to accomplish anything, in order to accomplish the process. It is presumed that resonance between the echoed acoustic stimulation and neural oscillations play a part mechanistically, which could be thought of like a musical instrument tuning itself.

Some sessions occurred with eyes closed, for which the participant was instructed to relax while sitting or reclining in a chair (Human Touch PC‐6). Some sessions occurred with eyes open, during with the subject could read, complete a word search, or just relax.

Those assigned to the NL group received randomly generated tones, in the context of sham‐HIRREM intervention sessions, as an active placebo. All activities, procedures, and sessions times were similar, with placement of sensors on various scalp locations. Sensors used for the NL group had no active recording function, and the tones were randomly generated with no relationship to current brain activity.

### 2.4 Data management

Study data were collected and managed using REDCap electronic data capture tools hosted at Wake Forest School of Medicine (Harris et al., 2009, 2019). REDCap (Research Electronic Data Capture) is a secure, web‐based software platform designed to support data capture for research studies, providing (a) an intuitive interface for validated data capture; (b) audit trails for tracking data manipulation and export procedures; (c) automated export procedures for seamless data downloads to common statistical packages; and (d) procedures for data integration and interoperability with external sources.

### 2.5 Outcome measures

A series of measures were collected at the enrollment visit, and also at three postintervention study visits (V1–V4), including self‐report symptom questionnaires, and continuous recordings of BP and HR, used to analyze for measures of autonomic cardiovascular regulation. An expectation measure regarding group assignment was obtained at V1 and V3, as well as following the 4th session during the intervention period. The second collection of the expectation measure during the intervention period, but prior to anticipated meaningful benefit in the LB group, allowed a realistic evaluation of the effectiveness of the blinding for the sham control intervention. Participants were also asked to maintain a daily sleep diary between V1 and V3. The primary outcome was differential change in the score reported on the Insomnia Severity Index (ISI) from V1 to V3.

### 2.6 Questionnaires

#### 2.6.1 Insomnia severity index

The severity of insomnia symptoms was measured using the Insomnia Severity Index (ISI) with each data collection visit. The ISI is a 7 question measure, with responses from 0 to 4 for each question, yielding scores ranging from 0 to 28 (Bastien, Vallieres, & Morin, 2001; Morin, Belleville, Belanger, & Ivers, 2011).

#### 2.6.2 Psychological and psycho‐physiological function

Depression was measured by the Beck Depression Inventory‐II (BDI‐II) (Beck, Steer, & Brown, 1996). If the participant indicated that he/she had suicidal thoughts or feelings, the PI was notified, and a protocol activated to ensure access to care if needed. Anxiety was measured by the Beck Anxiety Inventory (BAI) (Hewitt & Norton, 1993). Health‐related quality of life was measured by the EQ‐5D (Rabin & de Charro, 2001).

#### 2.6.3 Expectation measure

An expectation measure regarding the participant's impression about which intervention they were receiving was used to assess adequacy of blinding, as well as to explore potential effect of expectation on outcomes. This was a one‐item assessment inquiring, “Please guess as to which study group, Active HIRREM or Placebo, you are assigned.”

#### 2.6.4 Blood Pressure (BP), Heart Rate (HR), Heart Rate Variability (HRV), Baroreflex Sensitivity (BRS), and Blood Pressure Variability (BPV)

Continuous BP and HR were acquired from noninvasive finger arterial pressure measurements and ECG for a minimum of 10 min in subjects lying down quietly, supine. Systolic BP and beat‐to‐beat, RR, intervals (RRI) files generated via the data acquisition system (BIOPAC acquisition system and Acknowledge 4.2 software) at 1,000 Hz are analyzed using Nevrokard SA‐BRS software (Nevrokard BRS, Medistar) for measures of BRS, HRV, and BPV as follows: Frequency Method. Power spectral densities of SBP and RRI oscillations are computed by 512 points Fast Fourier Transform (FFT) and integrated over specified frequency ranges (LF: 0.04–0.15 Hz; HF: 0.15–0.4 Hz). A Hanning window was applied, and the squared‐coherence modulus is computed if coherence is >0.5 as reported. The square root of the ratio of RRI’s and SBP powers was computed to calculate LF, HF alpha indices, which reflect BRS. Power of RRI spectra in LF, HF range (LF_RRI_ and HF_RRI_) is calculated in normalized units, and the ratio of LF_RRI_/HF_RRI_ is used as a measure of sympathovagal balance.

The sequence method was used for calculation of BRS, based on quantification of sequences of at least three beats (n) in which SBP consecutively increases (UP sequence) or decreases (DOWN sequence), which are accompanied by changes in the same direction of the RRI of subsequent beats (n + 1). The software scans the RRI and SBP records, identifies sequences, and calculates linear correlation between RRI and SBP for each sequence. If the correlation coefficient exceeds a preset critical value (0.85), the regression coefficient (slope) is calculated and accepted. The mean of all individual regression coefficients (slopes), a measure of sequence BRS, was then calculated for Sequence UP, DOWN, and TOTAL. Time‐domain analysis included three time‐domain parameters to evaluate hemodynamic variability.

### 2.7 HRV data processing and interpretation

Heart rate variability was determined by computing the standard deviation of normal beat‐to‐beat interval (SDNN) and the root mean square of successive beat‐to‐beat differences in R‐R interval duration (rMSSD). BPV was determined as the standard deviation of the mean arterial pressure (SDMAP).

### 2.8 Blood pressure

Blood pressure (BP) measurements were taken with a finger cuff on two fingers of the left hand after calibration to brachial pressure while lying down on an examination table.

#### 2.8.1 Daily sleep diary

Participants were asked to maintain an online daily sleep diary. This measure was adapted from Carney et al. (2012) and has eleven questions to evaluate the quantity and quality of sleep (Table 2). The daily sleep diary allowed assessment of a variety of sleep parameters including sleep efficiency, total sleep time, sleep onset latency, wake time after sleep onset, sleep quality, and whether the participant felt rested and refreshed on awakening. Data collection of the daily sleep diary was accomplished via Internet data entry using the REDCap system. Participants were emailed a link to the online questionnaire and instructed to complete the sleep diary every day, preferably at the same time of day. Study staff reviewed response every few days and sent reminders if participants missed an entry for more than 3 days in a row. Paper diaries were provided in cases where participants had limited Internet access, or planned travel. For analysis, data from the week following the V1 visit and the week prior to V3 visit were compared. Those with at least 4 entries for both weeks were included for analysis.

**Table 2:**
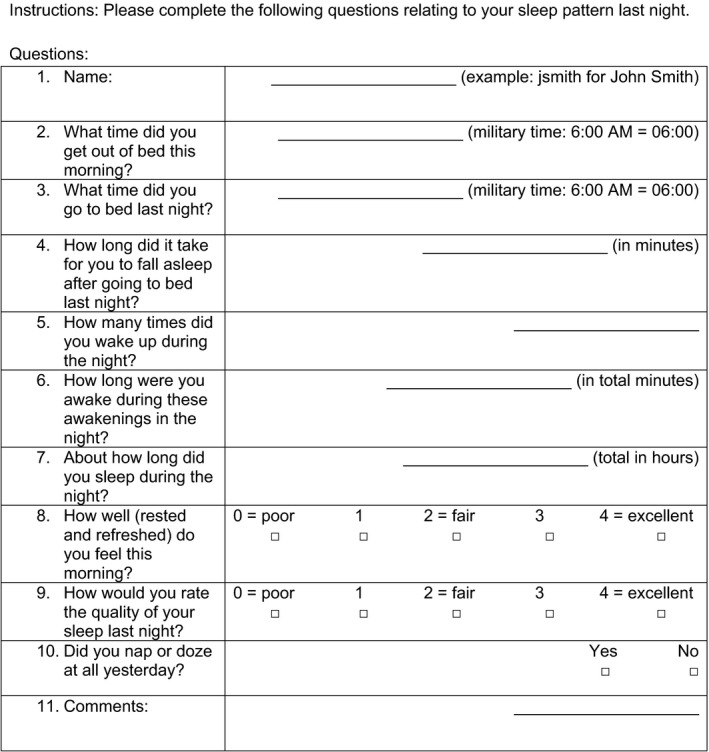
Daily sleep diary questions

#### 2.8.2 Safety and adverse events

Participants were asked about any new or worsened symptoms at every data collection visit, and during Technologist check‐ins prior to intervention sessions. Continuing reviews were submitted to the IRB annually.

### 2.9 Statistical analysis

Linear mixed models (LMMs) were used to contrast longitudinal changes in outcome measures between the LB and NL groups (Laird & Ware, 1982). LMMs provide a natural mechanism to address correlations induced by repeated measurements on a single subject as well as the likely presence of incomplete data due to participants that are lost to follow‐up. The primary analytic model included fixed effects corresponding to group assignment, measurement time point, and their interaction. Mean contrasts were used to compare the change for the outcome measures between groups from baseline to the follow‐up assessments at V2, V3 (our primary test of efficacy), and V4. Following recent practical guidelines for LMMs (Cheng, Edwards, Maldonado‐Molina, Komro, & Muller, 2010), we used a combination of goodness‐of‐fit measures (Edwards, Muller, Wolfinger, Qaqish, & Schabenberger, 2008), residual‐based diagnostics, and outcome transformations to address important assumptions (homogeneity of the variance and normality for the model residuals) and specification of the covariance structure. The LMMs were fitted using PROC GLIMMIX in SAS.

## 3 RESULTS

**FIGURE 1:**
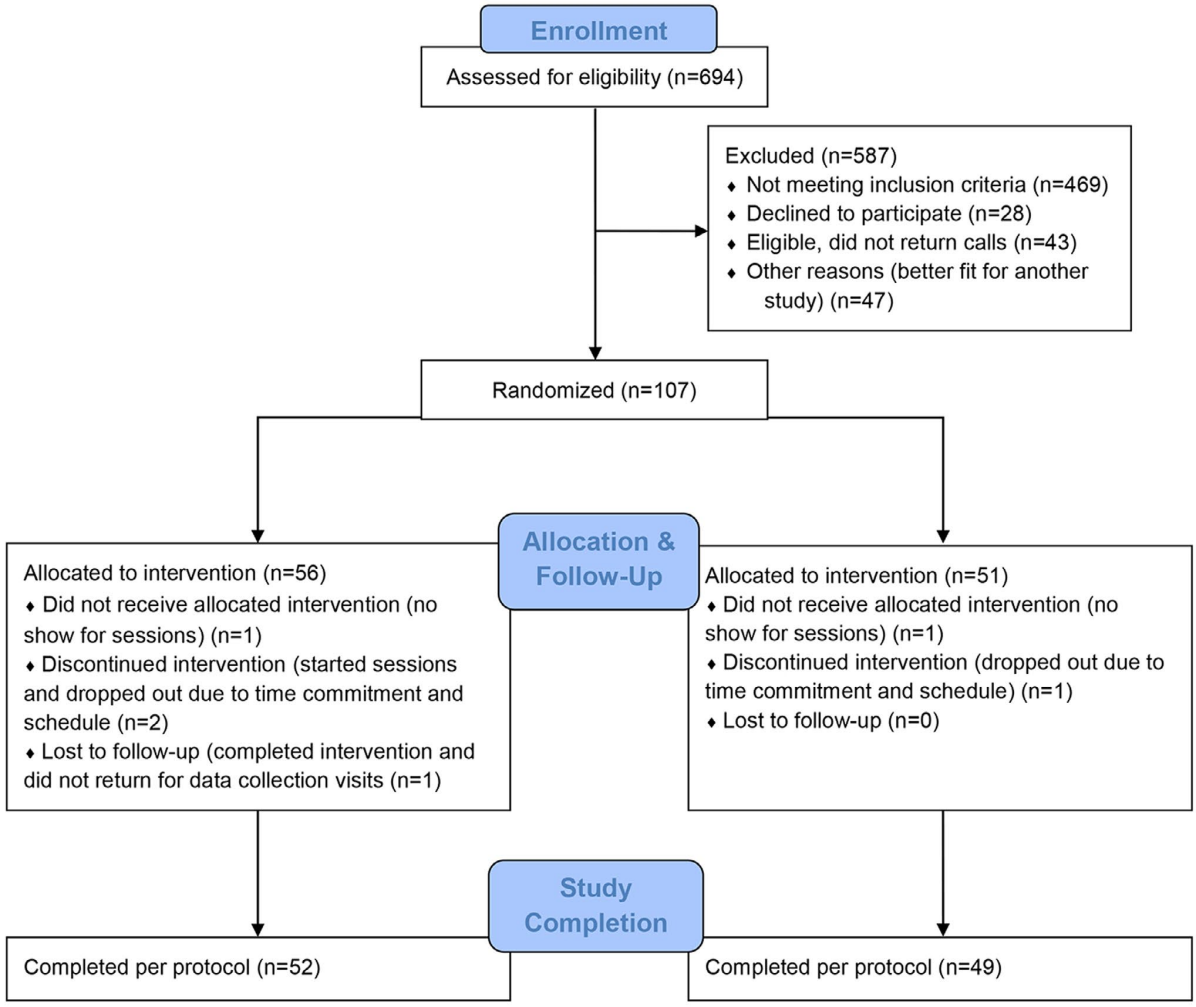
Consort diagram showing the flow of participants through the study for the groups receiving tones linked to brainwaves (LB), and tones not linked to brainwaves (NL)

Participant flow for screening, enrollment, randomization, and follow‐up of study subjects is shown in Figure 1. There were no statistically significant differences at baseline between the two intervention groups in terms of demographic or clinical characteristics (Table 1). The times over which the LB and NL interventions were received, and the intervals to data collection time points are outlined in Table 3. There were no important differences between groups for the total days to receive the intervention, in‐office seat number of days, days between V1 and the start of sessions, days between last session and V3, or V4.

**Table 3:** Data collection details

Variable (Mean and Standard deviation)	LB Group (*n* = 56)	NL Group (*n* = 51)
Days receiving intervention	7.0 (2.4)	7.2 (2.5)
In‐office days for intervention	5.5 (1.0)	5.6 (1.0)
Days between V1 and S1	9.0 (2.0)	10.0 (2.5)
Days between S10 and V2	6.0 (2.1)	6.0 (2.1)
Days between S10 and V3	62.0 (4.3)	62.0 (5.3)
Days between S10 and V4	119.0 (6.0)	120.0 (8.2)

Note

V1 is Visit 1, S1 is Session 1, S10 is Session 10, V2 is Visit 2, V3 is Visit 3, and V4 is Visit 4.

### 3.1 Participation and adequacy of blinding

A total of 107 enrolled participants were randomized (56 to LB, 51 to NL) and form the cohorts used for the intention‐to‐treat analyses. Of those assigned to LB, 53 completed the intervention (1 dropped out prior to sessions, and 2 discontinued interventions due to conflicts with time commitment and schedule). One who completed intervention did not return for data collection, yielding 52 who received the LB intervention per protocol. In the NL group, 49 completed the intervention (1 dropped out prior to sessions, and 1 discontinued intervention due to conflicts with time commitment and schedule). No NL participants were lost to follow‐up, so 49 were included for per protocol analysis of the primary outcome.

**FIGURE 2:**
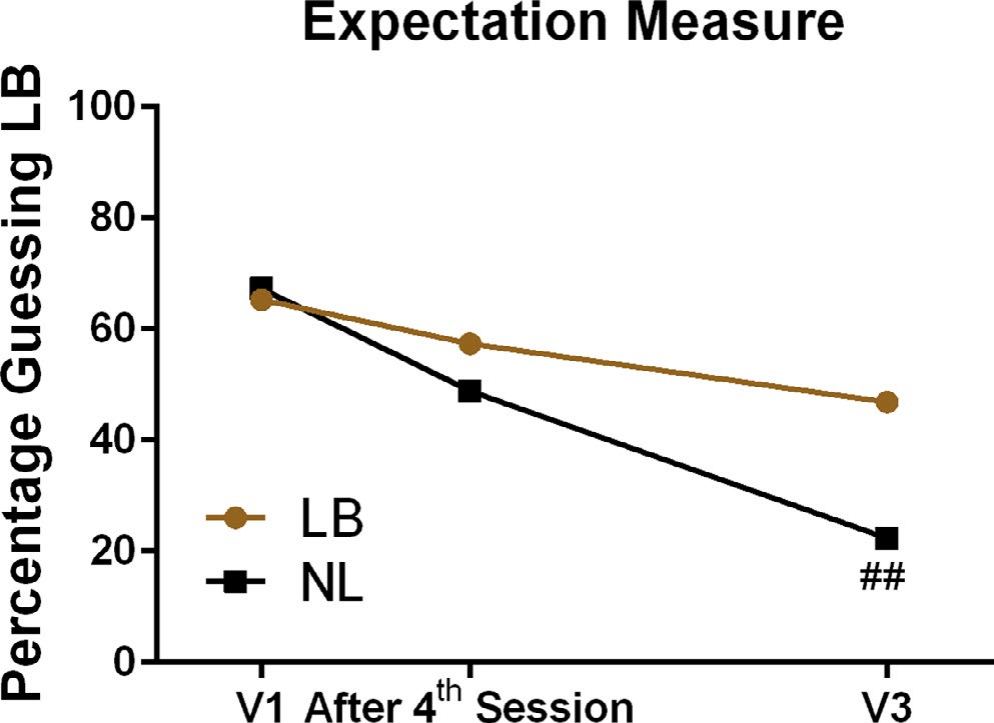
Expectation measure over time. Percentage of participants who guessed that they were in the group receiving tones linked to brainwaves (LB) compared to tones not linked (NL) to brainwaves (## = *p* < .01)

Based on the expectation measure at V1, and following session 4 during the intervention period, the sham procedures utilized to provide the tones not linked to brainwaves effectively blinded participants regarding group assignment (Figure 2). There were no important differences in baseline expectations (65.3% of LB and 67.3% of NL, *p* = .71). Blinding during the intervention period remained adequate until the expectation measure was repeated after the 4th session (57.4% of LB and 48.9% of NL, *p* = .40), but the reality of their total experience over time eventually tracked with group assignment. By V3, 8–10 weeks following intervention completion, 46.9% of LB, but only 22.4% of NL felt they had received HIRREM (*p* = .01).

### 3.2 Sleep outcomes

**FIGURE 3:**
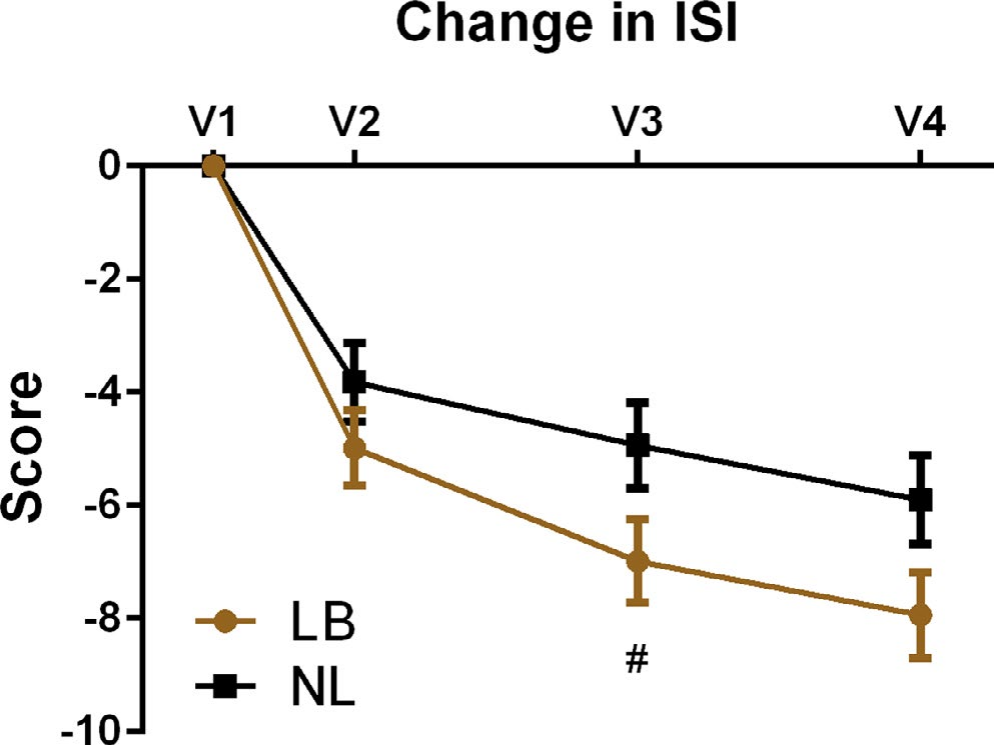
Intention‐to‐treat outcomes for the Insomnia Severity Index (ISI) at baseline (V1), 1–7 days (V2), 8–10 weeks (V3, primary outcome), and 16–18 weeks after intervention for those receiving tones linked to brainwaves (LB) compared to tones not linked (NL) to brainwaves (# = *p* < .05)

The primary outcome for this study was differential change in ISI scores from V1 to V3. Among all 107 randomized patients (intention‐to‐treat analysis using the GLIMMIX procedure, Figure 3), there was a mean reduction of ISI score in the NL group of −4.93 points (*SE* ± 0.76). There was an additional, significant reduction of −2.05 points (0.74) for those in the LB group (*p* = .045). Total ISI score reduction from V1 to V3 was −4.93 points in NL, with a reduction of −6.98 for LB. Additional reduction of −2.30 points (0.76) was still present in the LB group at V4 (*p* = .058). This marginally significant *p*‐value provides moderate evidence that the difference in symptoms still remained at V4. Total score reduction from V1 to V4 was −5.90 points for NL group, with a clinically meaningful reduction of −7.93 points in the LB group.

**FIGURE 4:**
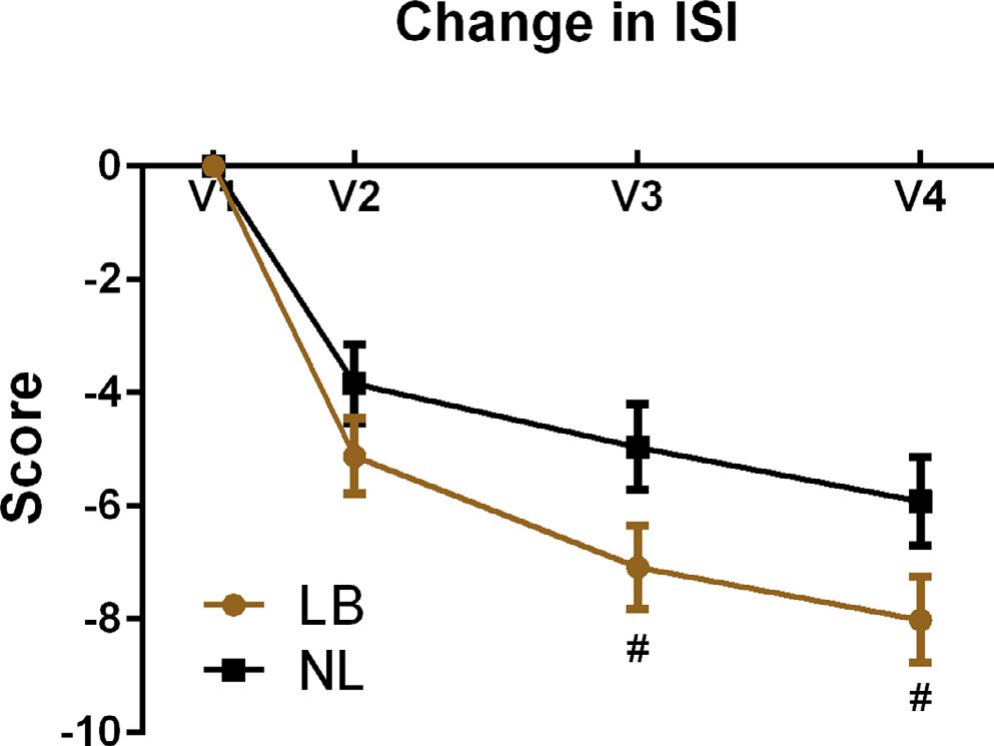
Per protocol ISI outcomes for the Insomnia Severity Index (ISI) at baseline (V1), 1–7 days (V2), 8–10 weeks (V3, primary outcome), and 16–18 weeks after intervention for those receiving tones linked to brainwaves (LB) compared to tones not linked (NL) to brainwaves (# = *p* < .05)

Among those who received the intervention per protocol (*n* = 101), analysis of change in ISI scores from V1 to V3 (Figure 4) showed a mean reduction of ISI score in the NL group of −4.96 points (0.76), with an additional significant reduction of −2.12 points (0.74) in the LB group (*p* = .038). Total ISI score reduction from V1 to V3 for per protocol analysis was −4.96 in NL, with −7.08 for LB. Additional reduction of −2.09 (0.76) points was still present in LB at V4 (*p* = .051). Total score reduction from V1 to V4 was −5.92 points for NL and −8.02 points for LB. There was no association in either group between key baseline characteristics (age, gender, or years of sleep trouble) and change in ISI score from V1 to V3. There was also no correlation between time of day (morning or afternoon) for the intervention or data collection, and ISI outcomes.

### 3.3 Other symptom outcomes

**FIGURE 5:**
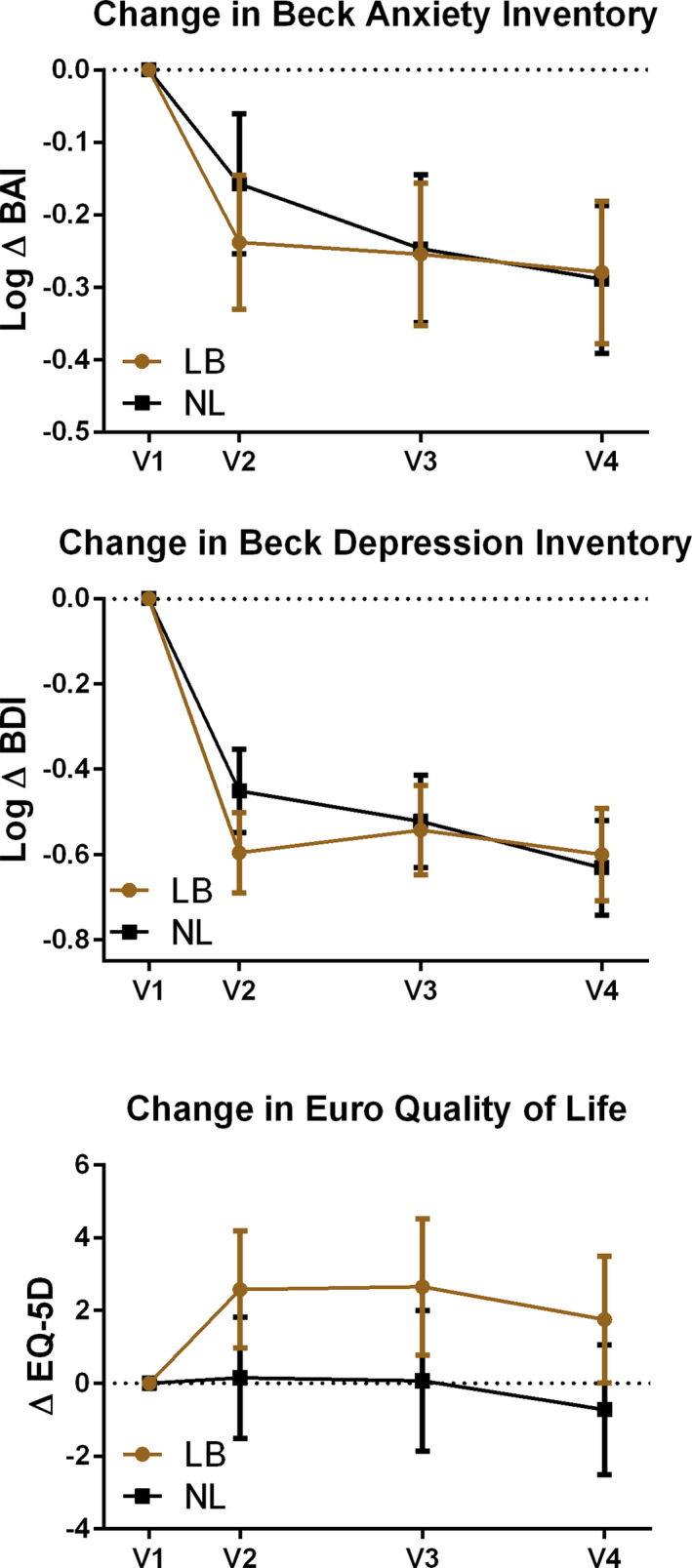
Panel of secondary self‐reported outcomes over time with Log change for Beck Anxiety Inventory and Beck Depression Inventory, and change in Euro Quality of Life score for those receiving tones linked to brainwaves (LB) compared to tones not linked (NL) to brainwaves

Baseline scores did not meet clinical criteria for depression or anxiety. Reduced symptoms were noted in both groups with self‐report symptom inventories for depression and anxiety (Figure 5). There was a trend for improved quality of life scores in the LB group.

### 3.4 Autonomic cardiovascular regulation

**FIGURE 6:**
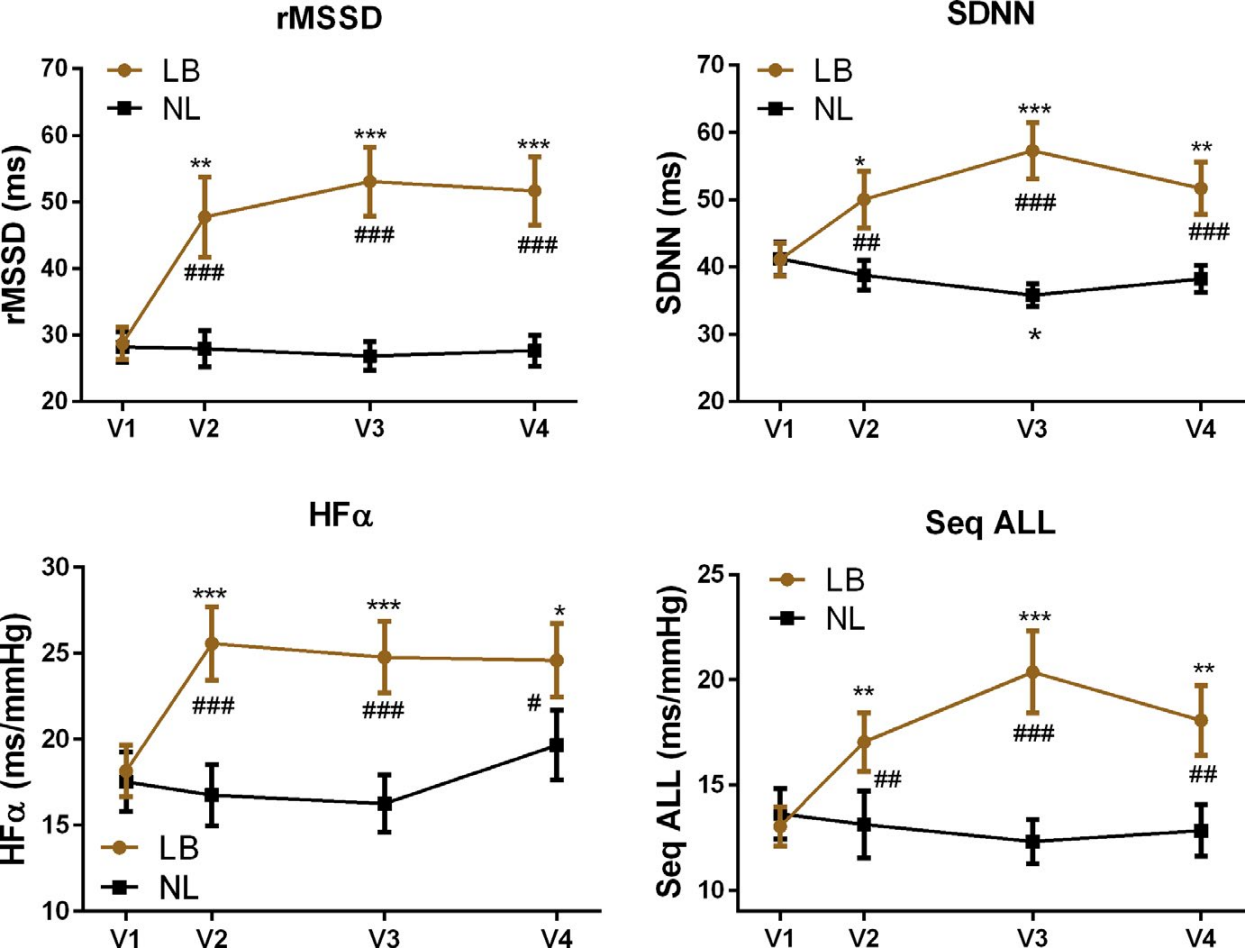
Panel of 4 autonomic outcome measures including rMSSD, SDNN, HF Alpha, and Sequence ALL for those receiving tones linked to brainwaves (LB) compared to tones not linked (NL) to brainwaves. rMSSD and SDNN reflect HRV and HF Alpha and Sequence ALL show changes in BRS. Results shown include change within groups over time (* = *p* < .05, ** = *p* < .01, *** = *p* < .001), and differences between groups (# = *p* < .05, ## = *p* < .01, ### = *p* < .001)

Based on intention‐to‐treat analysis, significant interval improvements were observed across multiple measures of HRV (SDNN and rMSSD) and BRS (HFα, and Sequence ALL) at all data collection time points (V2, V3, and V4), compared to V1 in the LB group (Figure 6). The improvements in the LB group were also significant when compared to outcomes in the NL group at all data collection time points. In the NL group, there was significant worsening of SDNN at V3, but no other significant changes were observed compared to V1 values.

### 3.5 Sleep diary outcomes

**FIGURE 7:**
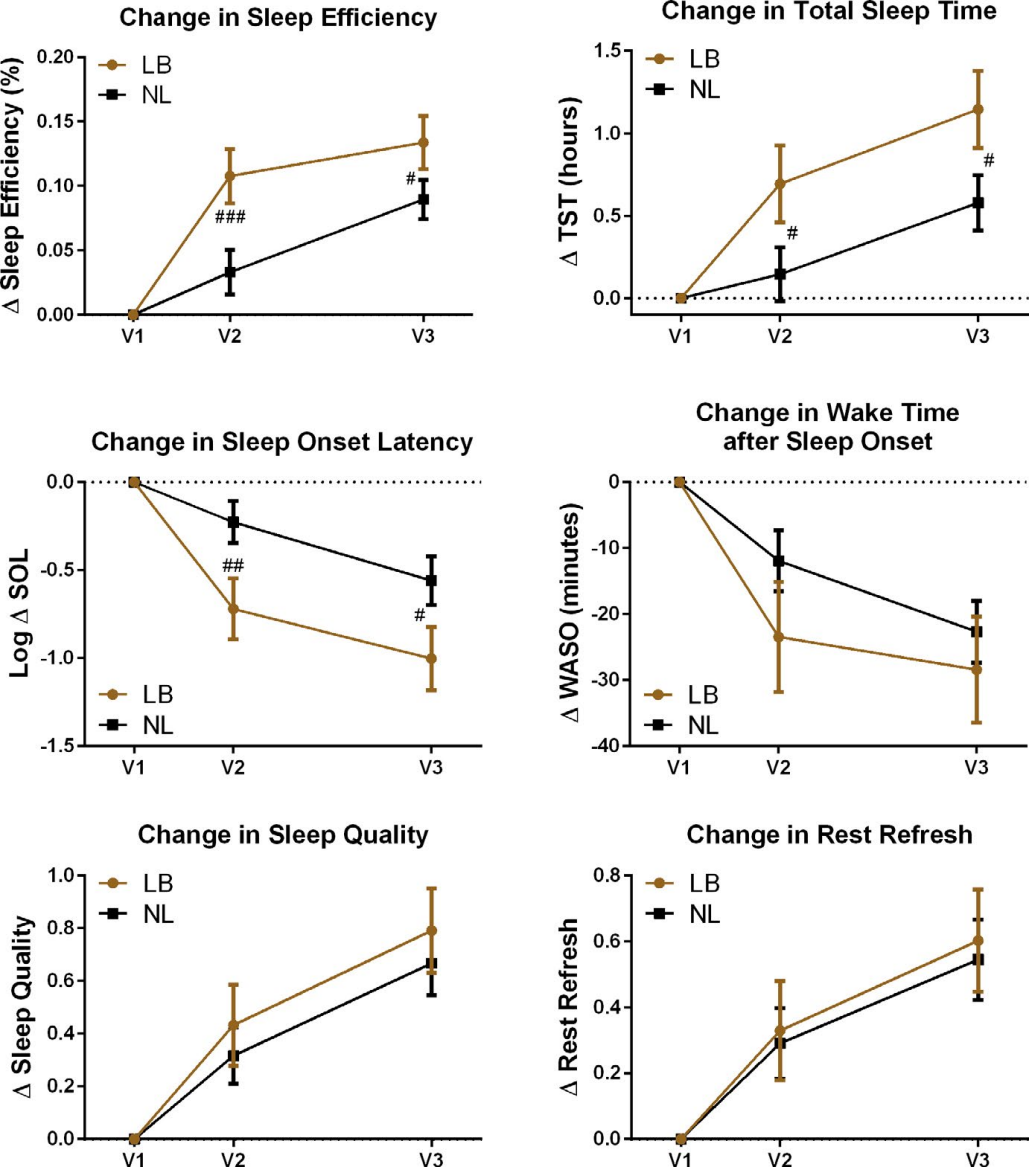
Panel of 6 sleep diary outcomes over time reporting differences between groups for those receiving tones linked to brainwaves (LB) compared to tones not linked (NL) to brainwaves (# = *p* < .05, ## = *p* < .01, ### = *p* < .001)

Sleep diary measures improved in both groups, compared to V1 data, but significant between‐group differences were observed at V2 and V3 for sleep efficiency, total sleep time, and sleep onset latency (Figure 7). Baseline sleep diary data were analyzed for correlation with ISI value at V1. Waking rested and refreshed (*r* = −.49, *p* < .0001), and sleep quality (*r* = −.54, *p* < .0001) correlated with the self‐reported insomnia t V1, while other sleep diary measures did not.

### 3.6 Safety and adverse events

There were few dropouts (5.6%), and no serious adverse events were reported. Nonserious, temporary, and somewhat paradoxical effects, that were judged to go beyond the intensity, expression, or nature of pre‐existing health conditions, were reported during study participation by 10.7% in the LB group and 13.7% in the NL group. Such symptoms included the participant reporting being more aware of, or more intensely affected by their feelings, or by those around them, changes in sleep, including dreams, emotions, energy levels, or a feeling of fullness in the head or mild headache. All episodes were brief, typically resolving in hours to 1–2 days, but at the most lasted <1 week. Skin irritation at the site from the paste used to affix the sensors to the scalp was reported by a single participant (<1%).

## 4 DISCUSSION

In this randomized, blinded, placebo‐controlled clinical trial of a novel closed‐loop neurotechnology, individuals with insomnia made ten visits entailing the receipt of acoustic stimulation within the context of a relaxed study setting. The intervention group of interest listened to audible tones of variable pitch and timing that were produced by software‐guided, algorithmic analysis of their real time brain electrical activity (HIRREM), while the placebo intervention group listened to nonspecific, randomly generated tones. After completion of their sessions and at follow‐up to 4 months, subjects in the HIRREM (LB) group reported reduced insomnia symptoms and also showed greater improvements in short‐term measures of autonomic cardiovascular regulation, than those who received random tones not linked to brainwaves (NL), as an active, sham placebo condition. The sham procedures resulted in effective blinding, with no significant differences in expectation measures regarding which intervention was being received, before or during the intervention. The magnitude of insomnia symptom reduction was clinically meaningful with HIRREM, but not placebo (Morin et al., 2011).

Sleep diary outcomes demonstrated added benefit between groups for sleep efficiency, total sleep time, and sleep onset latency. Although still reliant on self‐reporting, sleep diary outcomes are widely used to evaluate the impact of sleep interventions. These sleep diary data broaden the scope of the study outcomes and support to understanding of the primary results.

The present findings add to a growing body of literature indicating the utility of closed‐loop monitoring and acoustic stimulation as a meaningful way to impact sleep. Feasibility for modulation of sleep spindles through provision of audible tones in synchrony with slow oscillations has been shown during laboratory sleep in humans (Lázár, Dijk, & Lázár, 2019). To our knowledge, the present study is the first to report findings of a placebo‐controlled trial of a noninvasive, fully closed‐loop neurotechnology (i.e., one that monitors real time activity while requiring no conscious learning or volitional efforts for self‐modulation), for any health condition. These results, showing benefits for both symptoms and objective autonomic outcomes, obtained using a noninvasive, nonpharmacological approach, and with a relatively brief period of intervention, are thus unique and suggest value as an alternative approach for mitigating symptoms of primary insomnia.

We found it intriguing that the between‐group differences were more marked for the objective outcomes of autonomic cardiovascular regulation. Given that placebo effects may be more likely for subjective compared to objective measures (Schwarz & Büchel, 2015), this finding appears to indicate that interventional benefit for the LB group was due to the closed‐loop monitoring, algorithmic analysis, and acoustic stimulation linkage to brainwaves rather than subjective expectation, social support from the study team, or nonspecific effects of acoustic stimulation. This interpretation is buttressed by secondary analysis of a placebo‐controlled trial of vestibular therapy for insomnia, which found that differential change in HRV distinguished responders from nonresponders in the group receiving the primary intervention (Campana et al., 2011). Moreover, the HRV and BRS changes shown in the present study are consistent with the premise that successful allostatic therapeutics should be associated with healthful influence on peripheral (“downstream”) organ system dysregulation (Lee, Gerdes, Tegeler, Shaltout, & Tegeler, 2014; Sterling, 2012).

### 4.1 Limitations

Although HRV is an objective outcome measure, future studies of closed‐loop neurotechnology for insomnia may benefit from inclusion of other sleep‐specific measures such as actigraphy or polysomnography. This, although some uncertainty remains regarding interpretation of actigraphy in a subset of insomniacs who may lie awake, but immobile (Marino et al., 2013) scores for depression and anxiety did not meet clinical criteria at baseline, so a floor effect may have affected those outcomes. Subjects in this study had a mean age of >50 years, were mostly female, and of white, non‐Hispanic ethnicity. Greater diversity in future studies could help to inform generalizability. The use of several commonly prescribed categories of medications was also an exclusion. Other questions not addressed by this study include the implications of the present findings for individuals using psychotropic agents, the feasibility of use‐case scenarios involving medication discontinuation, and the potential applicability for other behavioral or health objectives. Future studies could include longer follow‐up periods and scheduled follow‐up intervention sessions to try to extend the period of benefit.

## 5 CONCLUSION

In conclusion, this randomized, controlled, clinical trial found that usage of HIRREM, a noninvasive, closed‐loop, allostatic, acoustic stimulation neurotechnology resulted in greater reductions of insomnia symptoms and improvements in autonomic cardiovascular regulation, than exposure to an active, sham placebo condition. The between‐group differences for objective physiological measures suggest that benefits were not likely due to a placebo effect or random chance, and they also raise the possibility for healthful effect on physical comorbidities associated with insomnia. Future studies are warranted to explore effects on other measures or mechanisms of sleep disturbance, and in other populations.

## CONFLICT OF INTEREST

All authors affiliated with the Wake Forest School of Medicine have no conflicts to report. Lee Gerdes, is currently, and Dr. Sung W. Lee, was previously, employed by Brain State Technologies, Scottsdale, AZ.

## AUTHOR CONTRIBUTIONS

CLT, HS, SWL, LG, and CHT involved in conception and study design, and interpretation and reporting of data for this project. CLT and HS involved in data acquisition. CLT, HS, SWL, SLS, and CHT prepared and analyzed the data. All authors have reviewed the manuscript and agreed to be accountable for all aspects of this work.

### Peer Review

The peer review history for this article is available at https://publons.com/publon/10.1002/brb3.1826.

## Data Availability

The data that support the findings of this study are available from the corresponding author upon reasonable request.
